# The relationship between 896A/G (rs4986790) polymorphism of *TLR4* and infectious diseases: A meta-analysis

**DOI:** 10.3389/fgene.2022.1045725

**Published:** 2022-11-24

**Authors:** Marcos Jessé Abrahão Silva, Davi Silva Santana, Letícia Gomes de Oliveira, Ellerson Oliveira Loureiro Monteiro, Luana Nepomuceno Gondim Costa Lima

**Affiliations:** ^1^ Evandro Chagas Institute (IEC), Ananindeua, Brazil; ^2^ Institute of Health Sciences (ICS), Federal University of Pará (UFPA), Belém, Brazil; ^3^ Department of Biomedicine, University of the Amazon (UNAMA), Ananindeua, Brazil

**Keywords:** immunogenetics, infectious diseases, association studies, single nucleotide polymorphism, toll-like receptor 4 (TLR4)

## Abstract

*Toll-like Receptors (TLRs)*, such as the *TLR4*, are genes encoding transmembrane receptors of the same name, which induce a pro- or anti-inflammatory response according to their expression as the host’s first line of defense against pathogens, such as infectious ones. Single nucleotide polymorphisms (SNPs) are the most common type of mutation in the human genome and can generate functional modification in genes. The aim of this article is to review in which infectious diseases there is an association of susceptibility or protection by the *TLR4* SNP rs4986790. A systematic review and meta-analysis of the literature was conducted in the Science Direct, PUBMED, MEDLINE, and SciELO databases between 2011 and 2021 based on the dominant genotypic model of this SNP for general and subgroup analysis of infectious agent type in random effect. Summary odds ratios (ORs) and corresponding 95% confidence intervals (CIs) were calculated for genotypic comparison. I^2^ statistics were calculated to assess the presence of heterogeneity between studies and funnel plots were inspected for indication of publication bias. A total of 27 articles were included, all in English. Among the results achieved, the categories of diseases that were most associated with the SNP studied were in decreasing order of number of articles: infections by bacteria (29.63%); caused by viruses (22.23%); urinary tract infection—UTI (7.4%), while 11 studies (40.74%) demonstrated a nonsignificant association. In this meta-analysis, a total of 5599 cases and 5871 controls were finalized. The present meta-analysis suggests that there is no significant association between TLR4-rs4986790 SNP and infections (OR = 1,11; 95% CI: 0,75–1,66; *p* = 0,59), but in the virus subgroup it was associated with a higher risk (OR = 2,16; 95% CI: 1,09–4,30; *p* = 0,03). The subgroups of bacteria and parasites did not show statistical significance (OR = 0,86; 95% CI: 0,56–1,30; *p* = 0,47, and no estimate of effects, respectively). Therefore, it has been shown that a diversity of infectious diseases is related to this polymorphism, either by susceptibility or even severity to them, and the receptor generated is also crucial for the generation of cell signaling pathways and immune response against pathogens.

## Introduction

Infectious diseases are the second leading cause of mortality worldwide and the main factor in generating disability-adjusted life years (DALYs), which a DALY corresponds to the loss of 1 year of healthy life (World Health Organization, 2000). They are diseases of great weight for health surveillance due to being associated with poverty and inappropriate living conditions (de Souza et al., 2020). The social, demographic, economic, and health control transformations that occurred between 1950 and the 2000s were extremely important for reducing the magnitude of various infectious diseases in several countries around the world. However, from the end of the 20th century, there was a behavior of reappearance of emerging diseases, such as polio, measles, tetanus, diphtheria, pertussis, tuberculosis, malaria, and acquired immunodeficiency virus (HIV/AIDS) disease and new diseases, for example, coronavirus disease 2019 (COVID-19) that counterbalanced this situation and took global proportions (Waldman et al., 1999).

In a population endemic to a disease, some individuals are diagnosed with active infection, which can generate unfavorable clinical outcomes and even death, while others remain asymptomatic (Janeway and Medzhitov, 2002). This can be explained by the distinct immunological response developed by each individual, which is largely correlated with his genetic background. Immunogenetic studies involving genetic background factors have become of great value in the evaluation of susceptibility or infection protection roles (Johnson et al., 2006; Doetschman, 2009).

Single nucleotide polymorphisms (SNPs) are the most frequent type of mutation in the human genome. They can serve as important biological markers for defining therapeutic, diagnostic, and prophylactic strategies for diseases, including infectious ones (Caetano, 2009). Thus, a study of strategies for prevention and control of the occurrence of diseases in humans through health promotion improves the quality of life of the community and contributes to new public health perspectives (Souza and Grundy, 2004). Epidemiological studies seek, in general, to analyze this occurrence of diseases and investigate control measures (Ishikawa and Gomide, 2019).

Toll-like Receptors (TLRs) are a family of type 1 transmembrane receptors and are evolutionarily characterized and conserved proteins in vertebrates and invertebrates (Medzhitov et al., 1997). In this way, TLR4 functions as a specific cell receptor for viruses, bacterial and fungal components, can act both on the cell surface and endosomes for recognition of the linker and to initiate activation of cytokine production by the innate immune response and conduction of the adaptive immune response (Reis et al., 2016). It is an important mediator in the detection of lipopolysaccharide (LPS) and manuronic acid polymers, both found in Gram-negative bacteria. LPS is a key factor in triggering systemic inflammatory response that can lead to sepsis, organ failure, and septic shock (Jabłońska et al., 2020). TLR4 also recognizes lipoteichoic acid (LTA) from Gram-positive bacteria (Takeuchi et al., 1999), mannans from fungal pathogens (Campos et al., 2004) and mycobacteria such as *Mycobacterium tuberculosis* (Sánchez et al., 2010). The physico-chemical properties and location of LTA in Gram-positive bacterial cells are similar to those of LPS in Gram-negative bacteria (Takeuchi et al., 1999).

TLR4 can be activated by oxidized phospholipids, which also appear in viral lung infections, such as in the detection of respiratory syncytial virus fusion protein (Kurt-Jones et al., 2000) and severe acute respiratory coronavirus syndrome 2 (SARS-CoV-2) (Aboudounya and Heads, 2021). Therefore, it activates the innate immune response (Khanmohammadi and Rezaei, 2021). Furthermore, it can identify certain molecular patterns associated with damage (DAMPs) generated by dead or lysed cells after host tissue injury or viral infection (Aboudounya and Heads, 2021).

**FIGURE 1:**
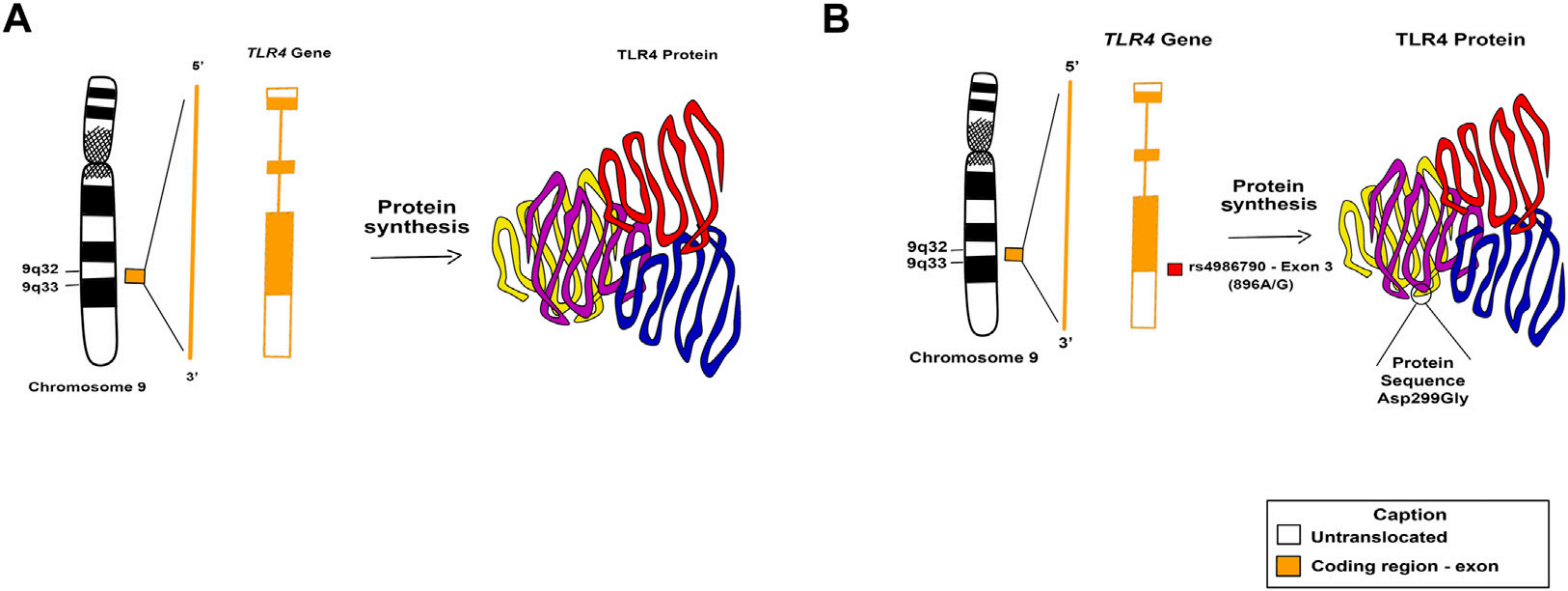
Location of the SNP rs4986790 on the *TLR4* gene. **(A)** Normal structure of the *TLR4* gene and protein. **(B)** SNP rs4986790 in the altered gene *TLR4* and protein structure.

The human *TLR4* gene is located on the human chromosome 9q33.1 and has three exons and two introns (Vaure and Liu, 2014). In this gene, SNP rs4986790, also known as 896A/G, is a non-synonymous mutation characterized by substitution within the 3rd exon of the *TLR4* gene, from an adenine (A) to a guanin (G) at position 896 (896A>G) which leads to modification of the conserved residue of aspartic acid to a glycine in amino acid 299 of the protein sequence (Asp299Gly) on, in or at the domain of the extracellular structure of TLR4. It is responsible for causing change in the function of the encoded protein, diminished function, i.e. *missense* (Ohto et al., 2012). It has already been associated with the susceptibility and protection of various infectious and non-infectious diseases, as well as the severity of some of them (Arbour et al., 2000). The frequency of the G allele of this SNP ranges from 0 to 20% among populations (Agnese et al., 2002). Figure 1 demonstrates the location of SNP rs4986790 in the *TLR4* gene and protein in a didactic model.

Due to their importance in generating an immune response in the fight against pathogens, polymorphic variants of TLR4, such as rs4986790, have great value in identifying the risk and defense of infectious diseases. In this sense, the following problem arises: “In which Infectious Diseases is the SNP rs4986790 proven to be related to susceptibility or protection?".

## Material and methods

### Identification and eligibility of relevant studies

This work consists of a systematic review, which uses the PICOS strategy for the elaboration of the guiding question in order to offer greater engendering of results and resolution of the problem, its anagram being characterized by: population; intervention; comparison; outcome; and study design (Santos et al., 2007). Thus, the following question was asked: “In which Infectious Diseases is the SNP rs4986790 proven to be related to susceptibility or protection?” Following the ensuing questions: Patient: patients with Infectious Diseases/Intervention: evaluate the *TLR4* SNP for each population studied and infectious diseases/Comparison: *TLR4* SNP and Infectious Diseases/Outcome: identification of which Infectious Diseases are associated with susceptibility or protection through the SNP studied/Study designs: cross-sectional studies, clinical trials, case-control and cohort studies.

The following keywords (MeSH) were selected as search strategy: “*TLR4*” and “Polymorphisms” and “Infectious Diseases”, together with the Boolean operator “AND”. The research was carried out in the following databases: National Library of Medicine National Institutes of Health of the United States (PubMed), Cochrane Collaboration and Medical Literature Analysis and Retrieval System Online (MEDLINE), Scientific Electronic Library Online (SciELO) and Science Direct.

Available, complete open access of the original categories in Portuguese, English, or Spanish, of the types of cross-sectional studies, clinical trials, case-control, and cohort studies were defined as inclusion criteria between 2011 and 2021. This period was used to condense updates on the subject over the last decade. Exclusion criteria were articles published prior to 2011, articles that were duplicated, available only abstract, letters to the editor, inaccessibility to important information in the article and articles with topics not pertinent to the research question. Thus, the final sample was reached, characterizing all stages, inclusion, and exclusion.

### Quality assessment and data extraction

Excel software was used for organization and screening of titles and abstracts and grade pro GDT software for classification of articles in the system. To classify the studies, the Grading of Recommendations Assessment, Development and Evaluation (GRADE system) was used, which is a system created to identify the degree of evidence and the strength of health recommendations (Aguayo-Albasini et al., 2014). For the inclusion of the articles, only those with high or moderate methodological quality were used.

### Synthesis of data and risk of bias

For the synthesis of data, the studies were presented according to the following categories: author, year, type of infectious agent, methodology, study population, genotyping method, allele-genotype frequencies in cases and controls, results of the Hardy-Weinberg test (HWE), country, Ethnic Group and results. Degrees of significance (*p* < 0.05) were considered statistically significant correlations between the SNP and each infectious disease studied. The Preferred Reporting Items for Systematic Reviews and Meta-Analyzes (PRISMA) flowchart, based on the PRISMA protocol, was used to present the steps followed for the present study (Liberati et al., 2009).

The data selection step for the visualization of the search was performed by two investigators (MJAS and DSS) independently, thus ensuring their reliability. Evidence is graded by research design, providing standards that establish the appropriate grade for medical decision making. The types of studies included in this article were composed according to the degree of evidence in decreasing order of clinical trials, cohort, case-control, and cross-sectional studies (Duncan and Schmidt, 1999).

### Statistical analysis

The meta-analysis was carried out using Review Manager 5.4.1 (Nordic Cochrane Center, Cochrane Collaboration, Copenhagen, Sweden). Only genotypic comparison was performed employing a dominant genetic model (AG + GG vs AA) was performed due to the rarity of the GG genotype (1% in some populations, such as East Asian) (Kim and Jeong, 2020). Summary odds ratios (ORs) and corresponding 95% confidence intervals (CIs) were determined using a random effect analysis method. A division into subgroups was prepared for evaluation by the types of infectious agents found here for this *TLR4* SNP. Heterogeneity between studies for comparisons was assessed using I^2^ statistics; A score of 25% indicates the absence of substantial heterogeneity, a result of 5% or more shows substantial heterogeneity, and a result of 75% or more indicates considerable heterogeneity. One of the most popular methods to determine the importance of heterogeneity is the chi-square test, with a conventional level of more conservative significance of *p* < 0.10, instead of the usual *p* < 0.05.

## Results

**FIGURE 2:**
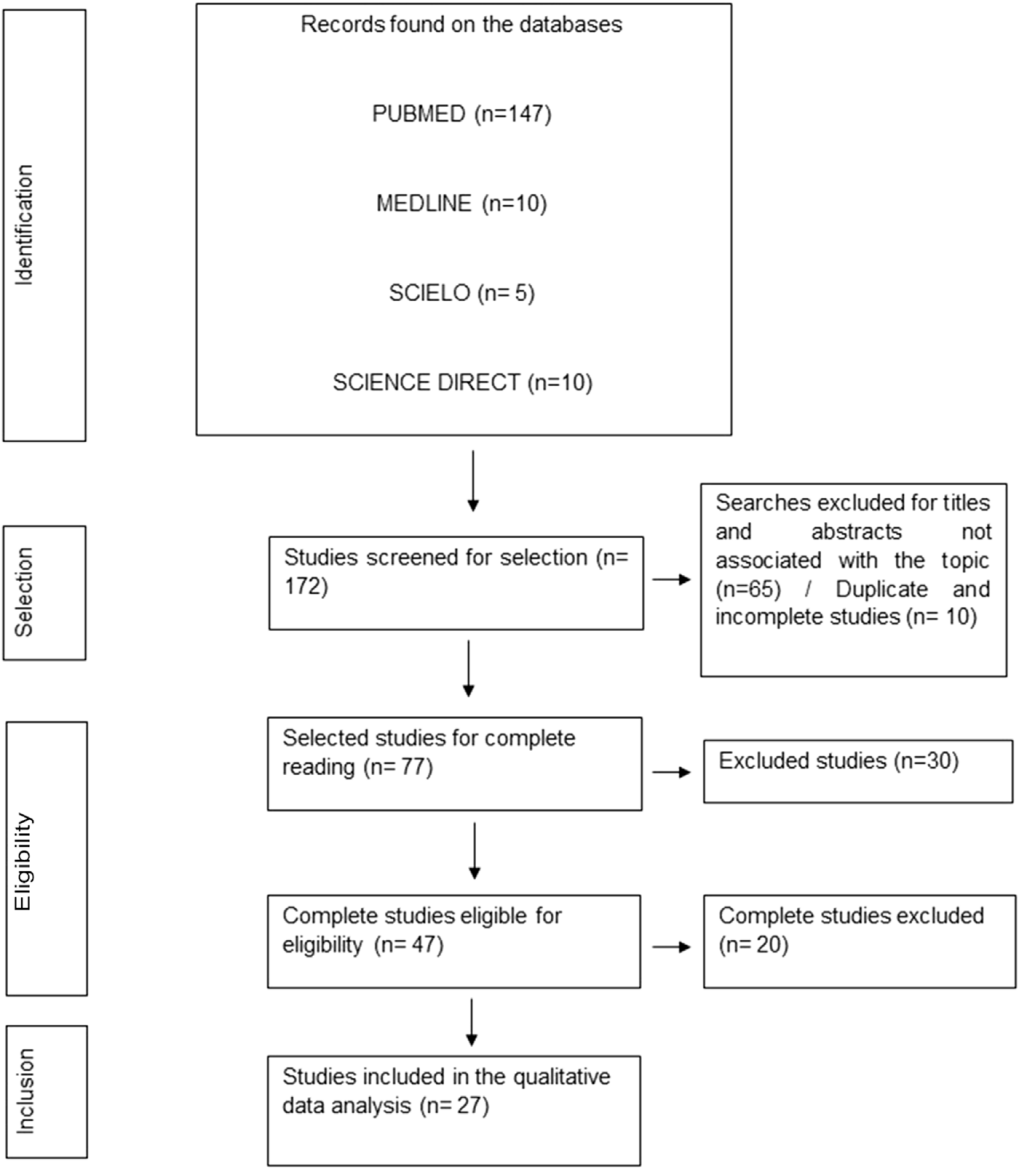
Flowchart representative of the stages of selection, eligibility, and inclusion of studies for analysis.

A total of 172 articles were found for reading from the databases. After excluding 10 duplicate studies, in addition to 20 letters to the editor, 20 studies available only the abstract, removing 94 studies irrelevant to the theme based on title, abstract, and body of the text. The final sample consisted of 27 articles, most of them belonging to the case-control type (Figure 2). These included studies are presented in Table 1. The presence of the mutant allele of the SNP was associated with susceptibility to several infectious diseases: COVID-19 (Taha et al., 2021); *Escherichia coli* infection (Sampath et al., 2013); *Orientia tsutsugamushi* infection (Janardhanan et al., 2013); *S. pyogenes* and *H. influenzae* (Liadaki et al., 2011); mononucleosis (Jabłońska et al., 2020); UTI in Indian diabetic patients (Gond et al., 2018); dengue infection (Sharma et al., 2016); influenza H1N1 in the Italian population (Esposito et al., 2012a); HBV/HCV (Sghaier et al., 2019); HIV (Vidyant et al., 2019).

**TABLE 1:** Characteristics of the studies included in the systematic review.

(Reference)	Disease/Type of infectious agent	Database	Methodology/Sample size	Genotyping method	Allele-genotypes frequencies (cases/Controls)	HWE	*p*-value	Country/Ethnic group	Results
Taha et al. (2021).	COVID-19/Virus	PUBMED	Cross-sectional study/300 individuals (150 adults with severe disease and 150 with not severe COVID-19)	PCR-restriction fragment length polymorphism (PCR-RFLP).	AA (76/117) AG (64/33) GG (10/0)	Not tested.	*p* < 0.001	Egypt/Not reported	The mutant allele G was associated with a significant positive risk of severe COVID-19. IL-6 levels, cytokine responsible primarily for the phenomenon of cytokine storms in patients, increased significantly with the presence of the mutant allele to reach the highest levels in the genotype GG (170 pg/ml (145–208.25))
Kresfelder et al. (2011).	Severe respiratory syncytial virus disease/Virus	PUBMED	Case-control/409 individuals (296 patients and 113 controls)	Fast Real-Time PCR	AA (265/99) AG (18/14) GG (0/0)	*p* > 0.05	*p* = 0,04	South Africa/African	The mutant allele of this SNP was associated with protection for severe respiratory syncytial virus disease
Emonts et al. (2011).	Rheumatoid arthritis/Bacteria	PUBMED	Case-control/839 individuals (376 patients and 463 controls)	PCR	AA (315/373) AG (54/58) GG (1/1)	*p* > 0.05	*p* = 0,43	Netherlands/White	No association was observed in this study for rheumatoid arthritis (RA)
Carroll et al. (2012).	Otitis media/Bacteria	PUBMED	Case-control/140 individuals (70 patients and 70 controls)	RT-PCR	AA (61/62) AG (9/8)	Not tested	*p* = 1	United States/White, Hispanic, African American, and Asian	No association with otitis media was observed
Esposito et al. (2012b).	Malaria/Parasite	PUBMED	Case-control/939 people (602 patients and 337 controls)	Fast Real-time PCR	AA (528/300) AG (72/36) GG (2/1)	*p* > 0,05	*p* = 0,55	Burundi/Not reported	No significant association between the presence of this SNP and malaria
Esposito et al. (2012a).	Influenza H1N1/Virus	PUBMED	Cohort study/436 individuals (272 with influenza-like illness (ILI), including 51 who were positive for pandemic A/H1N1/2009 virus, and 164 healthy controls)	Real-time PCR	H1N1-Influenza positive patient: AA (46/148) AG (5/16); H1N1-Influenza-negative patient: AA (202/148) AG (19/16)	*p* > 0,05	*p* = 0,51	Italy/White	The mutant allele of the SNP was associated with a higher risk of Influenza H1N1
Taha et al. (2014).	Rheumatoid arthritis/Bacteria	PUBMED	Case-control/30 individuals (20 patients with AR and 10 controls)	PCR Genotyping	AA (20/10) AG/GG (0/0)	Not tested	*p* > 0,05	Egypt/Not reported	No significant association was observed for RA
Sampath et al. (2013).	Gram-negative infections/Bacteria	PUBMED	Cohort study/408 individuals (318 controls and 90 patients)	Multiplexed single base extension (SBE) assay	AA (29/287); AG (10/31)	*p* > 0,05	*p* = 0.003	United States/Not reported	The mutant allele of the SNP has been associated with increased Gram-negative infections
Mrazek et al. (2013).	Prosthetic joint infection/Bacteria	PUBMED	Cohort study/539 individuals, subdivided into 350 patients and 189 controls	qRT-PCR	AA (318/166) AG (32/41) GG (0/1)	*p* > 0.05	*p* = 0.06	Czech Republic/White	The mutant allele was associated with a lower risk of developing prosthetic joint infection (PJI)
Sharma et al. (2016).	Dengue/Virus	PUBMED	Case-control/320 individuals (120 patients and 200 controls)	PCR Genotyping	AA (81/157) AG (37/42); GG (2/1)	*p* > 0,05	*p* = 0.042	India/Not reported	Carriers of the mutant G allele of this SNP were characterized as susceptible to dengue infection
Golovkin et al. (2015).	Endocarditis/Bacteria	PUBMED	Case-control/410 individuals (110 patients and 300 controls)	Real-time PCR	AA (95/253) AG (13/46) GG (1/1)	*p* > 0.05	*p* = 0,57	Russia/White	No association was observed between this SNP and infectious endocarditis
Torres-García et al. (2013).	Tuberculosis/Bacteria	PUBMED	Case-control/180 subjects, divided in 90 patients and 90 controls	PCR Genotyping	AA (88/89) AG (2/1)	*p* > 0.05	*p* > 0,05	Mexico/Mazatecan	No significant association was found between this SNP and tuberculosis
Sellers et al. (2016).	Periodontitis/Bacteria	PUBMED	Cohort study/617 participants, including 511 patients with rheumatoid arthritis or osteoarthritis and 105 controls	qPCR	Moderate periodontitis: AA (295/93) AG (41/12) GG (1/0); Severe periodontitis: AA (151/93) AG (22/12) GG (1/0)	*p* > 0,05	*p* = 0.045	United States/African-American and White	The mutant allele of this SNP interacted significantly *with P. gingivalis* in reducing the risk of periodontitis and may be protective against loss of alveolar bone height (ABHL), a characteristic of periodontitis. The interaction between SNP TLR4 and smoking was not significant in relation to periodontitis or ABHL
Sghaier et al. (2019).	Hepatitis B and C/Virus	PUBMED	Case control/634 individuals (274 patients, subdivided into 174 with HCV and 100 with HBV and 360 controls).	PCR-RFLP	AA (47/26) AG and GG (201/107)	*p* > 0,05	*p* < 0.001	Tunisia/Not reported.	The G allele was associated with a significantly increased risk of chronic HBV/HCV infection. Additionally, the GG genotype was positively associated with HBV-linked hepatocellular carcinoma (HBV)
Varzari et al. (2019).	Tuberculosis/Bacteria	PUBMED and Science Direct	Case-control/523 subjects (272 patients and 251 healthy controls)	Mass spectrometry	AA (121/228) AG (11/15) GG (1/0)	*p* > 0,05	*p* = 0,42	Moldova/Not reported	SNP was not significantly associated with susceptibility to *Mycobacterium tuberculosis* infection after correction of multiple tests
Akil et al. (2012).	Urinary Tract Infection (UTI)/Bacteria	PUBMED	Case-control/205 individuals (112 patients and 93 controls)	PCR Genotyping	UTI-Control Groups: AA (97/79) AG (14/14) GG (1/0); Upper-Lower UTI groups: AA (64/39) AG (13/1); Scar (+/-) Pyelonephritis group: AA (31/33) AG (9/4)	Not tested	UTI-Control Groups: *p* = 0,05;	Turkey/Not reported	The mutant allele was numerically elevated in patients with lower UTI and controls
Castro-Alarcón et al. (2019).	*E. coli* infection/Bacteria	PUBMED	Case-control/200 individuals (100 patients and 100 controls)	PCR-RFLP	Infected-Control Groups: AA (96/97)AG (4/3); *E. coli* Virulence Groups (hlyA): AA (13/83) AG (2/2) Symptomatology Groups: AA (37/59) AG (4/0)	*p* = 0,87	Infected-Control Groups: *p* = 0,7; Virulence Groups (hlyA): *p* = 0,04; Symptomatology Groups: *p* < 0,05	Mexico/Mestizo	The mutant allele of the SNP was associated with the risk of developing UTI and the virulence factor hlyA of *E. coli*, but not with susceptibility
Hussein et al. (2018).	Renal parenchyma infection/Bacteria	PUBMED	Case-control/510 individuals (380 patients and 200 controls)	PCR Genotyping	APN-Controls Groups: AA (84/188) AG (11/12) GG (3/0)	*p* > 0,05	*p* = 0.002	Egypt/Not reported	The mutant allele of the SNP was associated with decreased renal parenchyma infection instead of renal scar
Gond et al. (2018).	UTI/Bacteria	PUBMED	Case-control/1100 individuals categorized into four groups: Group I (diabetic patients with UTI, *n* = 318), Group II (diabetic patients without UTI, *n* = 324) Group III (non-diabetics with UTI, *n* = 200) and Group IV (healthy control matched with age, *n* = 260)	PCR-RFLP	Group I-Controls: AA (218/211); AG (95/47) GG (5/2) Group III-Controls: AA (141/211) AG (53/47) GG (6/2)	*p* > 0,05	Group I-Controls: *p* = 0,0009; Group III-Controls: 0.008	India/Not reported	It was revealed that the genotype A/G and the G allele of *TLR4* rs4986790 are significantly associated with UTI in diabetic and non-diabetic patients compared to the healthy control
Li et al. (2019).	Periodontitis/Bacteria	PUBMED	Cohort study/241 subjects subdivided into 164 patients and 77 controls	Mass spectrometry	AA (164/77) AG (0/0) GG (0/0)	Not tested	Not calculated	China/Not reported	This SNP has not been associated with periodontitis
Jabłońska et al. (2020).	Epstein-Barr Virus (EBV)/Virus	PUBMED	Clinical trial/289 subjects (149 patients and 140 controls)	PCR-RFLP	AA (128/130) AG (11/9) GG (10/1)	*p* > 0,05	*p* = 0.003	Poland/Not reported	The heterozygous genotype of SNP was associated with an increased risk of high levels of liver enzymes and leukocytosis. It revealed that it appears to be related to the course of acute Epstein-Barr Virus (EBV) infection in children and adolescents
Janardhanan et al. (2013).	*Orientia tsutsugamushi* infection/Bacteria	PUBMED	Case-control/271 people (137 patients with typhus and 134 controls)	PCR-RFLP	AA (103/116) AG (33/16) GG (1/2)	*p* > 0,05	*p* = 0.028	India/Tamil-speaking Dravidian	The mutant allele G was associated with susceptibility to *Orientia tsutsugamushi* infection. Compared to controls, the prevalence of the heterozygous SNP genotype was significantly higher among patients with exfoliating typhus
Vidyant et al. (2019).	Human Immunodeficiency Virus-1/Virus	PUBMED	Case-control/430 individuals, divided into 160 HIV-1 seropositive patients, who were subdivided according to the severity of the disease based on CD4 count (stages I, II and III), and 270 healthy seronegative subjects	PCR-RFLP	AA (120/234) AG (37/34) GG (3/2)	*p* > 0,05	*p* = 0.003	India/Not reported	The presence of mutant allele of the SNP is a risk factor in the susceptibility to HIV-1
Lee et al. (2011).	Gram-negative infection/Bacteria	PUBMED	Cohort study/706 patients, 108 with microbiologically confirmed gram-negative bacterial infections, 135 with clinically suspected but unconfirmed infections, and 463 patients without gram-negative bacterial infections	PCR Genotyping	AA (103/431) AG/GG (5/32)	Not tested	*p* = 0,39	United States/Not reported	A significant association was observed for a decrease in the risk of Gram-negative infections
Liadaki et al. (2011).	Tonsil-Tonsil disease/Bacteria	PUBMED	Case-control/327 individuals (289 controls and 38 patients)	PCR-RFLP	Patient-Control Groups: AA (30/24) AG (8/25) GG (0/0)	*p* > 0,05	*p* = 0.038	Greece/Not reported	The G allele provided a potential risk of Tonsil-Tonsil disease by *S. pyogenes* and *H. influenzae*
Garlet et al. (2012).	Periodontitis and Gingivitis/Bacteria	PUBMED	Case control/414 individuals (217 healthy controls—H and 390 patients, subdivided into 197 Chronic periodontitis - CP and 193—Chronic gingivitis - CG)	Real-time PCR	H/CP Groups: AA (135/131) AG (56/74) GG (6/12); H/CG: AA (105/131) AG (97/74); GG (13/12)	*p* > 0,05	H/CP: Groups: *p* = 0.0678; H/CG Groups: *p* = 0.0431	Brazil/White; Afro-American and Admixed	The mutant allele of the SNP was associated wit protection for chronic periodontitis and gingivitis.
Sahingur et al. (2011).	Periodontitis/Bacteria	PUBMED	Case-control/191 individuals (114 patients and 77 controls)	PCR and Taq Man Assay	AA (95/59) AG (19/17) GG (0/1)	*p* > 0,05	*p* = 0,25	United States/Not reported	No significant association with chronic periodontitis

The presence of mutant allele of this SNP was associated with the protection of: *Escherichia coli* infection (Lee et al., 2011); severe respiratory syncytial virus disease (Kresfelder et al., 2011); urinary tract-UTI infection (Akil et al., 2012); prosthetic joint infection in the Czech population (Mrazek et al., 2013); renal parenchyma infection (Hussein et al., 2018); chronic periodontitis and chronic gengivitis (Garlet et al., 2012); *P. gingivalis* infection (Sellers et al., 2016) and with the reduction in severity of periodontitis (Sellers et al., 2016).

**FIGURE 3:**
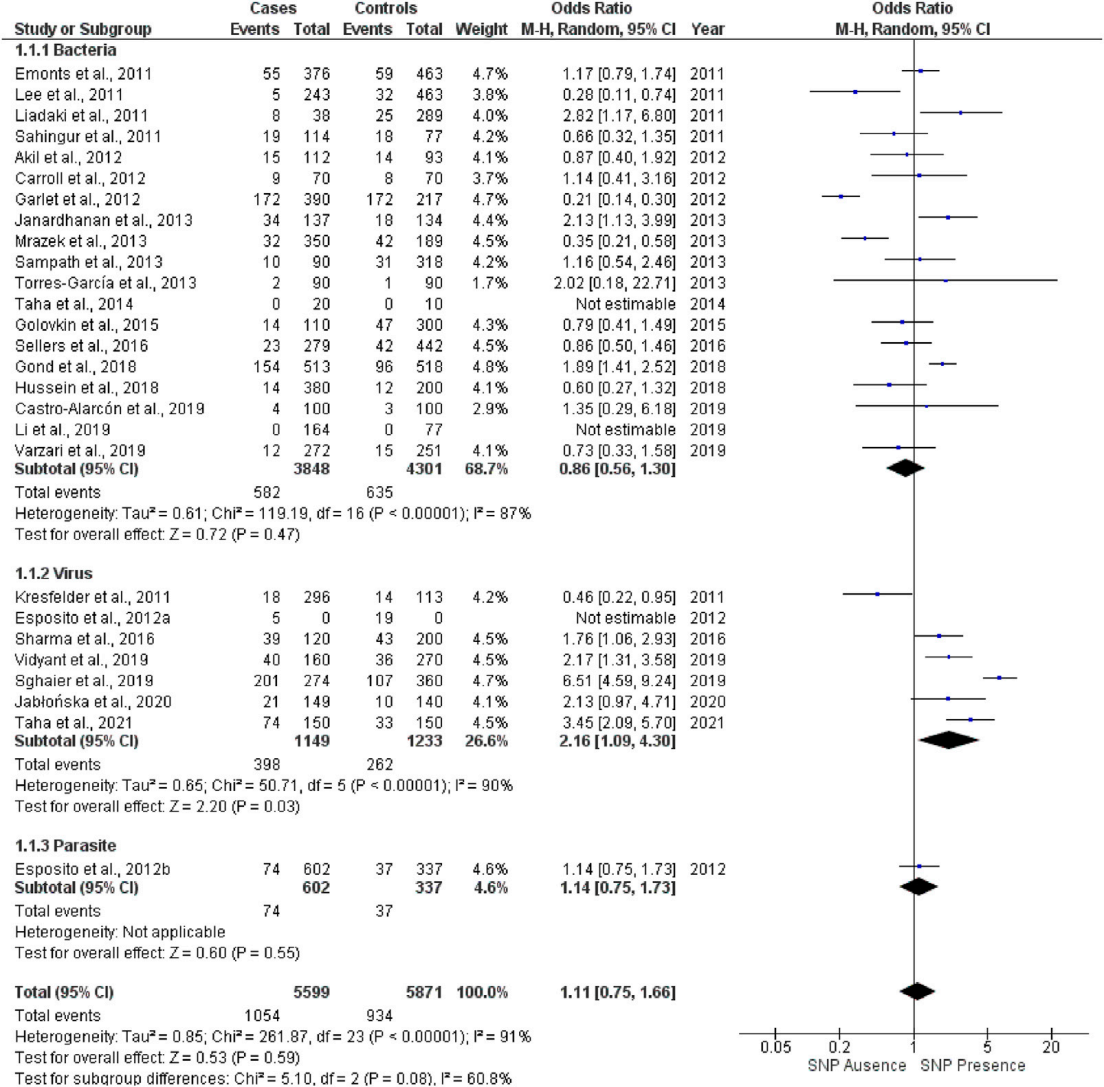
Forest plot of the association between the SNP rs4986790 of the *TLR4* gene and susceptibility to infectious diseases in a dominant genotypic model.

The forest plot of the meta-analysis was represented by Figure 3. The meta-analysis was performed for random effect due to the high heterogeneity found (I^2^) in the analysis of both subgroups and in general.

In a generalized analysis, the SNP *TLR4* rs4986790 did not show a significant association with susceptibility to infections (OR = 1,11; 95% CI: 0,75–1,66; *p* = 0,59). Regarding the subgroups, for bacteria, although this SNP was not statistically significant, it showed a greater strength of association for the protection of infections related to them (OR = 0,86; 95% CI: 0,56–1,30; *p* = 0,47). It should be taken into account that, in the meta-analysis, the etiological agent of UTI considered was bacteria. Regarding viruses, a significantly positive relationship was found between the presence of this mutant SNP allele and the risk of contracting viral infections (OR = 2,16; 95% CI: 1,09–4,30; *p* = 0,03). For parasites, only one study was included, and therefore it was not possible to observe significant associations in this subgroup (no effect estimate).

**FIGURE 4:**
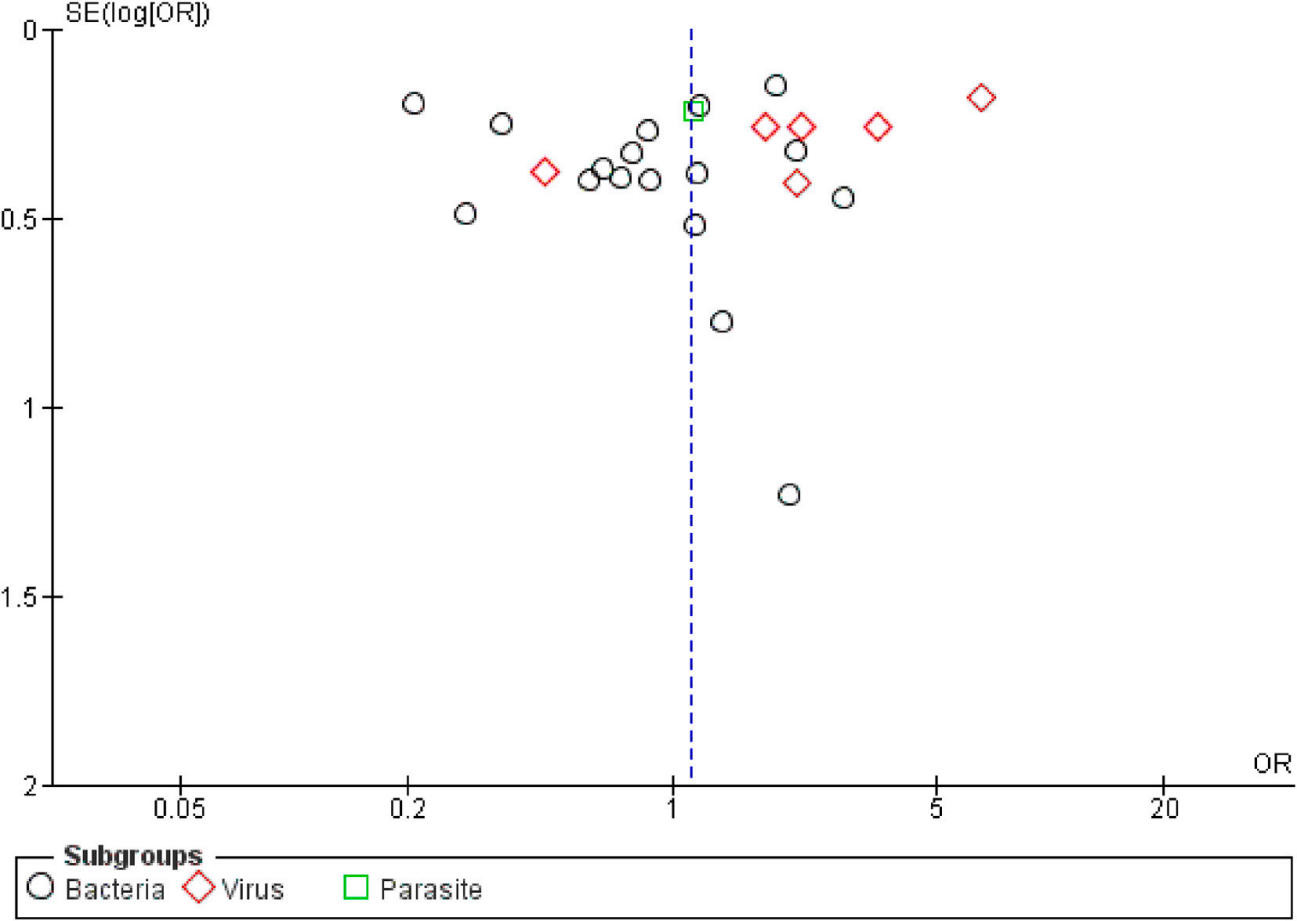
Begg’s funnel plot for the meta-analysis of the rs4986790 SNP of the *TLR4* gene.

A total of 5599 cases and 5871 controls were included. The numerical configuration of researches was composed of 19 studies (70.37%) for the bacteria subgroup, with 3848 cases and 4301 controls, while six researches (22.22%) were in virus subgroup (constituted of 1149 cases and 1233 controls) and only one for parasites (3.7%), forming a subgroup with 74 patients and 37 controls. A symmetrical funnel plot presented the reduced risk of bias in Figure 4.

**FIGURE 5:**
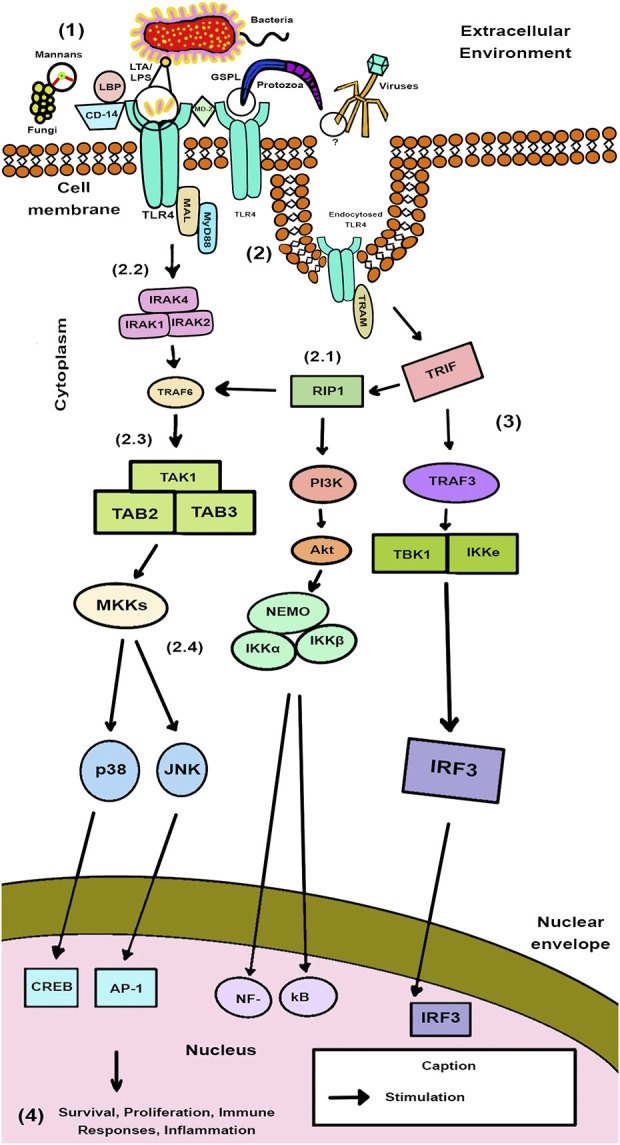
Schematic Model of TLR4 Signaling Transduction Pathways in Pathogen Recognition. Black circles from the top section of the figure indicate the PAMPs that are recognized by TLR4 for each type of infectious agent.

**FIGURE 6:**
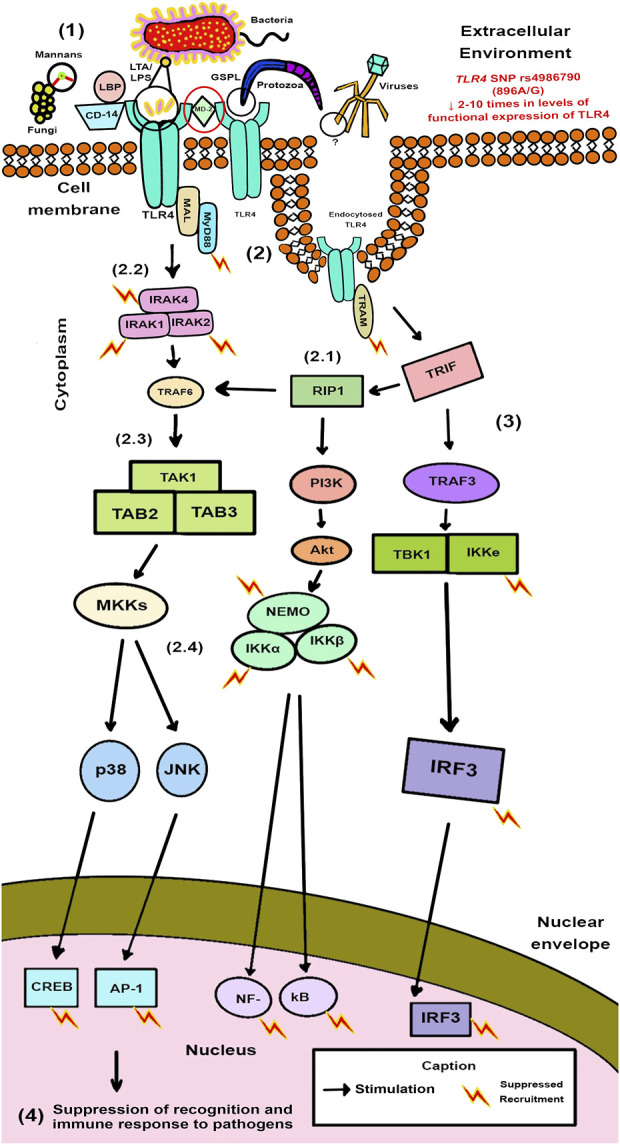
Schematic Model of TLR4 Signaling Transduction Pathways in Pathogen Recognition in the SNP rs4986790 presence. Black circles from the top section of the figure indicate the PAMPs that are recognized by TLR4 for each type of infectious agent. The red circle highlights the increase in receptor dysfunction in its immune functions in the absence of MD-2. The red lightning symbols indicate the structural components of the TLR4 signaling pathway in which the mutation was associated with increased infection sensitivity identified in humans.

Schematic presentation of the data found for TLR4 Signaling Transduction Pathways was performed in Figure 5 and these pathways in the presence of the investigated SNP of *TLR4* in Figure 6.

Figure 5 represented its stages chronologically through numbers in ascending order. It starts at phase (1) Recognition of the microorganism: TLR4 is activated on the cell membrane by PAMPs of pathogens, such as lipoteichoic acid (LTA) from Gram-positive bacteria, LPS from Gram-negative bacteria, elements not fully unraveled for viruses, fungal mannans, and glycosphingolipid (GSPL) from parasites, while CD-14, liposaccharide binding protein (LBP) and MD-2 act as accessory proteins in a conformational complex. From this binding, in phase (2), the left and right routes for TLR4 signaling are shown to be MyD88-dependent and independent, respectively, in which there is the generation of pathways: TLR4 dimerizes and recruits downstream adapter proteins such as MyD88/MAL and TRIF/TRAM to mount an inflammatory response in the cytoplasm. (2.1) RIP1 is recruited to the receptor by binding to TRIF and is then phosphorylated and polyubiquitinated, inducing Akt phosphorylation. (2.2) Concurrently, the MyD88 molecule-dependent activations are facilitated with IRAK4, which contributes to the phosphorylation of IRAK1. This reaction allows the binding of the TRAF6 molecule to the complex. (2.3) Furthermore, binding of TRAF6 activates TAK1, which forms an aggregation with TAB2 and TAB3. After its formation, TAK1 induces the IKK complex through NEMOylation. (2.4) Then, both pathways converge in the generation of NF-kB. In addition to activating NF-κB, TAK1 also phosphorylates MKKs, which subsequently activate JUN N-terminal kinase (JNK) and p38 to further enhance the inflammatory response. In stage (3) the TRIF/TRAM pathway not only activates NF-κB (translocated to the nucleus), but also triggers IRF3 to mount an antiviral response by interferons (IFNs) actions. In step 4) TLR4 levels cause the expression of pro-inflammatory cytokines and the maturation of APCs of the innate immune system that will define the course of the infection. This stimulation of TLR4 is crucial for inducing powerful responses to protect against host damage. Innate immunity develops because of the coordinated effects of activating several TLRs, together with the activation of numerous additional receptors involved in host defense and is linked to the formation of adaptive immunity. Specific defense is made possible by cytokine induction; for example, IRF-3 facilitates antiviral responses *via* IFN-β, while interleukin (IL) - 12 is crucial for defense against intracellular infections. TLR-mediated up-regulation of inducible NO synthase contributes to macrophages’ ability to destroy phagocytosed bacteria.

Figure 6 represents at phase (1) Recognition of the microorganism: TLR4 is activated on the cell membrane by PAMPs of pathogens, such as lipoteichoic acid (LTA) from Gram-positive bacteria, LPS from Gram-negative bacteria, elements not fully unraveled for viruses, fungal mannans, and glycosphingolipid (GSPL) from parasites, while CD-14, liposaccharide binding protein (LBP) and MD-2 act as accessory proteins in a conformational complex. *TLR4* mutation change the structure of *TLR4* and limit its ability to interact with LPS, CD-14, and/or MD-2. The *TLR4* SNP rs4986790 acts by decreasing the expression of TLR4 by 2 times. In this sense, the absence of myeloid differentiation factor 2 (MD-2), which forms a complex with TLR4 and LPS, this decrease is elevated in 10 times. From this binding, in phase (2), both MyD88-dependent and independent routes are affected by the presence of this mutation and there is the generation of TLR4 signaling pathways: the aforementioned vias (2.1), (2.2), (2.3), and (2.4). This mutation leads to suppressed recruitment of downstream adapter molecules and kinases involved in the TLR4 pathways, such as IRAK4, NEMO, IKKe and IRF3, which can thus eliminate signaling activity. In stage (3), there is suppression of all these vias, including, the NF-κB transactivation and translocation in human monocytes with the 896A/G *TLR4* expression, which could lead to the development of adaptive immunity. In step (4), this dysfunctional stimulation of TLR4 is characterized by suppression of recognition and immune response to pathogens, generating an uncoordinated immune response and, consequently, in most cases, a poor prognosis to infectious diseases.

## Discussion

The analysis of host genetic factors such as SNPs can become a useful strategy to identify people at increased risk of specific infections, patients at higher risk of unfavorable disease outcomes, and contribute to more effective therapeutic interventions (Mukherjee et al., 2019). The pro-inflammatory response induced by TLRs is considered the first form of body defense against pathogens and marks substantial variations in antimicrobial defense of populations.

In this sense, the manifestation of infectious diseases may be related to mutations in the *TLR4* gene due to the absence/presence of the mutant allele of SNPs, in addition to genetic, epigenetic, and environmental factors (Medzhitov and Janeway, 2000). SNP rs4986790 has been shown to cause hyporesponsiveness to LPS. This SNP causes local structural changes around the mutation area that can affect the effectiveness of folding, cell surface expression, protein stability, and interaction with downstream messenger proteins (Ohto et al., 2012). At the molecular level, this SNP has been reported to interfere with the interaction of TLR4 with *downstream messengers*, such as MyD88 and other downstream messengers (Figueroa et al., 2012). Additionally, SNP appears to affect the levels of functional expression of *TLR4*, causing a decrease of 2 times (Prohinar et al., 2010). This reduction is further amplified to 10 times in the absence of myeloid differentiation factor 2 (MD-2) that forms a complex with TLR4 and LPS (Park et al., 2009). This decline in functionality is further amplified before TLR4 and LPS form a complex (Prohinar et al., 2010).

In the present systematic review, most of the studies were observed to come from populations in the United States (5 studies - 18.51%) and the number of studies associated with susceptibility to infectious diseases was higher for the African continent (with 10 studies in all continents −37.03%). Despite the numerical supply of articles and the number of positive associations relating bacterial infections to the present SNP of *TLR4*, the strength of association of the meta-analysis did not generate a significant degree of due evidence from the analyzed researches, while for viruses there was a significant association. A total of 16 of the 27 studies (59.25%) did not report the ethnic group belonging to the investigated population, most of them coming from the Asian and African continent (5 researches each, with 31.25%) and, with respect to the country, mostly from the United States (with four researches, 25%). Therefore, there is a great bias in reporting these ethnic terms in these studies, especially from the United States.

In our analysis, the most studied diseases for SNP were those caused by bacterial infections (with 17 studies—62.96%), followed by viral infections, with seven studies, equivalent to 25.93% and urinary tract infection (UTI) with two studies (7.4%). The excess percentage was delimited by studies involving parasitic agents (1 study, 3.7%).

The most commonly associated infectious diseases in different countries for *TLR4* SNP and susceptibility were those caused by viruses (6 researches, 85.71% in virus subgroup). The infectious diseases that for this SNP were most associated with protection in different countries had several studies in decreasing order among those caused by bacteria infections (*n* = 9, 33.33%), viruses (*n* = 1, 3.7%), and UTI (*n* = 1, 3.7%).

Contradictory findings were found for susceptibility related to the presence of the mutant allele of this SNP and infectious diseases were found in populations around the world and this may be directly related to environmental or even epigenetic factors, associated with the individual genetic background. Conflicting data were found in different populations for the following diseases: reduced risk of periodontitis in Brazilian population (Garlet et al., 2012), and a protective effect was conferred for periodontitis in a United States population (Sellers et al., 2016), while no association between this SNP and periodontitis was found in populations in China and United States (Sahingur et al., 2011; Li et al., 2019); what concerns gram-negative bacteria infection, otitis media has no association with this SNP in the American population (Carroll et al., 2012), a no significant association was observed for susceptibility to *E. coli* infection in Mexico population (Castro-Alarcón et al., 2019); a protective effect conferred by this SNP in the United States population (Lee et al., 2011), a higher risk of these infections in Greece, India, and United States populations (Liadaki et al., 2011; Janardhanan et al., 2013; Sampath et al., 2013); regarding to UTI, the study conducted by Akil et al., 2012 associated the presence of the mutant allele of this SNP with reduced risk of this disease in Turkey (Akil et al., 2012), while Gond et al., 2018 revealed a higher risk of this infection in India (Gond et al., 2018).

No significant statistical associations were observed for the diseases: periodontitis (in the American and Chinese population) (Sahingur et al., 2011; Li et al., 2019); malaria in the Burundi population (Esposito et al., 2012b); tuberculosis (in Moldavia and Mexico populations) (Torres-García et al., 2013; Varzari et al., 2019); endocarditis in the Russian population (Golovkin et al., 2015); rheumatoid arthritis (in Egyptian population, Netherlands) (Emonts et al., 2011; Taha et al., 2014); otitis media in the American population (Carroll et al., 2012); *E. coli* infection in Mexico population (Castro-Alarcón et al., 2019).

### SNP rs4986790 in the *TLR4* gene in bacterial infections

The main association found in this review in a numerical offer for susceptibility to SNP and infectious diseases is correlated with the main TLR4 ligand (LPS) and reveals the effect of the G allele in various functional studies for Gram-negative bacteria (Jabłońska et al., 2020).

The functional expression of *TLR4* activation and its main ligand (LPS) emphasizes its activity in detecting the molecular patterns associated with pathogens (PAMPs) of Gram-negative bacteria (Jabłońska et al., 2020). The activation of TLR4 by LPS is a multistage process. The first step highlights the LPS binding protein (LBP), which extracts LPS from bacterial membranes and vesicles containing LPS and transfers it to the grouping of TLR4 differentiation 14 coreceptors (CD-14). CD-14 exists in two forms, soluble and membrane-bound. Both forms can interact with LPS-loaded LBP. CD-14 disassembles LPS aggregates and transfers monomer LPS to a hydrophobic pouch in myeloid differentiation factor 2 (MD-2), which is part of the MD-2/TLR4 complex. Activation of TLR4 leads to the recruitment of intracellular adaptor proteins, primary myeloid differentiation response 88 (MyD88) and/or toll/interleukin-1 receptor (TIR) containing interferon-beta (TRIF), leading to the expression and secretion of pro-inflammatory mediators (Kuzmich et al., 2017).

### SNP rs4986790 in the *TLR4* gene in viral infections

Viral infections have in common membrane-associated TLR4 activating ligands, which bind to the TLR4 complex. In this context, this structure is activated by non-LPS DAMPs and PAMPs, which vary widely in their structure—some without structural similarities with LPS, the determination of the role of TLR4 activation remains uncertain for viruses (Olejnik et al., 2018). Further studies can improve the understanding of the association of the role of this *TLR4* SNP in its receptor and how this activation can interfere with the recognition of viruses in human organisms, since a strong association was found between viruses and this SNP.

### SNP rs4986790 in the *TLR4* gene in urinary tract infection

UTI is an infectious disease that can be caused by bacteria, viruses, parasites, and fungi. Microorganisms enter through two pathways, the lower urinary tract or the bloodstream (into the kidneys). TLR4 has been reported to control the immune response to infection of epithelial cells that line the mucosa of the urinary tract and the signaling response of this TLR regulates cell balance and death by releasing some urothelium cytokines (Ruch and Engel, 2017). Finally, the UTIs were also considered in this study a disease of infection caused by Gram-negative bacteria due to their etiological diversity, even though in most studies of the polymorphism in question there was a predominance of infection by these bacteria. TLR4 signaling is activated by binding the P fimbriae to the glycosphingolipid (GSPL) receptor of the cell surface, and ceramide acts as a signaling intermediate to activate TLR4. In a type of human inoculation therapy, p-fimbriae have been shown to promote bacteriuria and mucosal inflammation. In addition, the *TLR4* SNP demonstrates importance in the innate immune regulation of UTI. In this scenario, more than 85% of UTIs are caused by bacteria and about 80% of them are caused by *Escherichia coli*. In addition to being caused by other Gram-negative bacteria and to a lesser extent by Gram-positive bacteria (Ragnarsdóttir et al., 2010).

In a review and meta-analysis by Reza Mortazavi-Tabatabaei et al., 2019, with epidemiological studies involving patterns of antibacterial resistance to UTI, among the most common pathogens that cause UTIs were *E. coli* (with about 80%)*,* followed *by Klebsiella, Staphylococcus*, and *Streptococcus* with a frequency of 62%, 13%, 12%, and 11%, respectively (Reza Mortazavi-Tabatabaei et al., 2019).

Based on a review and meta-analysis by Huang; Xu (2019), in Asian populations, the mutation in rs4986790 may make them more susceptible to urinary tract infections (Huang et al., 2020). This information on the association of the susceptibility to UTI for Asian populations was corroborated in the present review.

In the murine UTI model, TLR4 controls the innate immune response to *Escherichia coli* and TLR4 mice −/− develop asymptomatic bacteriuria instead of severe infection. Low levels of *TLR4* expression have also been observed in children with asymptomatic bacteriuria. In addition to being related to susceptibility and protection agaisnt UTI, the SNP can distinguish phenotypes from symptomatic and asymptomatic patients, it determines the severity of acute infection, as well as the long-term effects on the integrity of the renal tissue. On the other hand, susceptibility to UTI can also be discussed in terms of social and environmental factors or dysfunctional urination (Ragnarsdóttir et al., 2007).

### SNP rs4986790 in the *TLR4* gene in parasitic infections

As far as parasites are concerned, the highlighted SNP was only analyzed for malaria (in one study), with no significant association. At the molecular level, this receptor can function as an upstream sensor for GSPL and induce the intracellular inflammatory signaling necessary to kill parasites (Aguirre-García et al., 2019).

### SNP rs4986790 in the *TLR4* gene in fungal infections

With regard to fungi, no study had significant associations for the SNP analyzed. On the other hand, TLR4 has a scientific framework directly related to fungal infections, such as candidiasis and paracoccidioidomycosis, caused, respectively, by the fungi *Candida albicans* and *Paracoccidioides brasiliensis*. In this sense, it directly protects the oral mucosa from fungal infection by Candida through a process mediated by polymorphonuclear leukocytes (PMNs) (Qin et al., 2016). What concerns *P. brasiliensis*, its most virulent strain (Pb 18 strain) can use TLR4 to gain access to neutrophils that pathogenically produce interleukin (IL)—eight and IL-10, increasing the inflammatory process and thus patient morbidity (Acorci-Valério et al., 2010; Freitas et al., 2016).

### TLR4 signaling pathways in the immune response to pathogens and final considerations

SNPs can be associated or involved, or they can even determine the differential risks of infection. This is due to the fact that in some infections the immune response leads to a protective inflammatory response, while in others this response may be essential in the pathogenesis of the infectious process (Silva et al., 2021).

At this conjuncture, TLR4 signaling is mediated by four adapting molecules: myeloid differentiation factor 88 (MyD88); adapter-type protein MyD88 (Mal), also known as the TIR domain adaptor protein (TIRAP); TIR-containing ligant-induced interferon-beta (TRIF), also known as TLR-containing ligant molecule 1 (TICAM-1); TRIF-related ligant molecule (TRAM), also known as IRR-containing protein (TIRP) or ligating molecule 2 (TICAM-2). TLR4 requires all four ligands to mediate a comprehensive immune response (Lizundia et al., 2008).

MyD88 propagates through the factor associated with tumor necrosis factor receptor—TNF (TRAF)—6, which induces the production of an inflammation regulator, the nuclear factor - kappa beta (NF-κB). This regulator triggers the genes encoding the immune activators, which include tumor necrosis factor alpha (TNF-α) and interleukins (IL) -1, IL-8, IL-12, and IL-6. However, uncontrolled defense processes of organisms against infectious agents can lead to a number of organic dysfunctions, including coagulation dysfunction, fever, vasodilation, and decreased blood pressure, leading to necrosis of tissues and organs caused by the accumulation of cytokines (Costalonga and Zell, 2007).

As in a review and meta-analysis by Ziakas et al. (2013), this analysis highlights that this SNP of *TLR4* was associated with susceptibility to a diverse spectrum of infections and has a complex effect of the TLR4 variant on susceptibility to infectious diseases. However, a greater association with parasitic diseases is recorded in a study by Ziakas et al. (2013), while in this present study it is associated with viral infections. This is due to the fact that the aforementioned study sought to achieve two *TLR4* SNPs, with a different time frame, different types of epidemiological studies and only open access studies (Ziakas et al., 2013).

The presence of significant effects may depend on the magnitude of *TLR4* functional expression. Protection or risk can be reduced by the levels of functional expression of *TLR4*, which are regulated by its polymorphisms and the presence of MD-2. Therefore, it is necessary to explore whether MD-2 is important in response to some infections, but not others, or if TLR4 levels differ in one infection compared to another (Prohinar et al., 2010).

The limitations of the study are, in fact, the difficulty of its reproducibility due to the design, sample size, and environmental and genetic heterogeneity of the different populations around the world due to the diversity in genetic background. The environmental heterogeneity of populations is related to the various outcomes of diseases due to individual risk factors for each of them. In addition, different populations generate different immune responses that may affect conclusive information. Although this was true, the papers supported substantial contributions to answer the leading issue and featured current validation and standardization, enabling a crucial and effective qualitative synthesis of the data. Therefore, these elements do not suggest a considerable amount of methodological variability.

## Conclusion

The data engendered in this study indicate the importance of the main TLR4 (LPS) linker for SNP rs4986790 and infectious agents, since the most associated and studied infectious diseases were infections by Gram-negative bacteria, mediated by LPS, even with nonsignificant association here. This meta-analysis also indicates a new association between infections and this *TLR4* SNP with respect to viruses, with 2 times greater risk in this subgroup in a relationship of increased susceptibility to the presence of the mutant allele in individuals from different countries that needs to be further investigated.

In the same way that there are controversial results on susceptibility to infectious diseases, we report the need for immunogenetic studies that are performed in different populations. Furthermore, the study reveals the characterization of pathophysiology in clinical conditions of diseases associated with this SNP. From this perspective, new omic studies can help close the gaps in understanding the extent to which immunogenetics interferes with the outcome of infectious diseases. Therefore, this study provides an overview for the creation of strategies in the field of medicine with respect to the treatment of infectious diseases.

## Data Availability

The original contributions presented in the study are included in the article/Supplementary Material, further inquiries can be directed to the corresponding author.
